# Effects of multi-resistant *ScALDH21* transgenic cotton on soil microbial communities

**DOI:** 10.3389/frmbi.2023.1248384

**Published:** 2023-10-03

**Authors:** Qilin Yang, Jiancheng Wang, Dawei Zhang, Hui Feng, Tohir A. Bozorov, Honglan Yang, Daoyuan Zhang

**Affiliations:** ^1^ State Key Laboratory of Desert and Oasis Ecology, Xinjiang Institute of Ecology and Geography, Chinese Academy of Sciences, Urumqi, China; ^2^ Key Laboratory of Ecological Safety and Sustainable Development in Arid Lands, Xinjiang Institute of Ecology and Geography, Chinese Academy of Sciences, Urumqi, China; ^3^ University of Chinese Academy of Sciences, Beijing, China; ^4^ Turpan Eremophytes Botanical Garden, Chinese Academy of Sciences, Turpan, China; ^5^ Research Institute of Economic Crops, Xinjiang Academy of Agricultural Sciences, Urumqi, China; ^6^ State Key Laboratory of Biocontrol, School of Life Sciences, Sun Yat-sen University, Guangzhou, China; ^7^ Guangdong Provincial Key Laboratory of Plant Resources, School of Life Sciences, Sun Yat-sen University, Guangzhou, China; ^8^ Laboratory of Molecular and Biochemical Genetics, Institute of Genetics and Plants Experimental Biology, Uzbek Academy of Sciences, Tashkent Region, Uzbekistan

**Keywords:** environmental safety assessment, metagenome analysis, microbial community diversity, *ScALDH21*, soil microbial communities, transgenic cotton

## Abstract

Transgenic crops are increasingly prevalent worldwide, and evaluating their impact on soil microbial communities is a critical aspect of upholding environmental safety. Our previous research demonstrated that overexpression of *ScALDH21* from desiccant-tolerant moss, *Syntrichia caninervis*, in cotton revealed multi-resistance to drought, salt, and biotic stresses. We conducted metabarcoding using high-throughput sequencing to evaluate the effect of *ScALDH21* transgenic cotton on soil microbial communities. We further conducted soil tests to analyze the chemical properties of transgenic and non-transgenic cotton, including the total content and availability of chemical elements (K, P, and N), organic matter, and pH value. Both transgenic and non-transgenic cotton fields exhibited soil pH values higher than 8. The presence of transgenic cotton significantly enhanced the availability of available K and the total content of total P in the soil. Alpha and beta diversity indices of soil microbiota showed no difference between two transgenic and non-transgenic cotton groups. Dominant clades of fungal and bacterial genera were equivalent at the phylum and genus levels in all three groups. The correlation analysis of microbial communities and soil environmental factors revealed the absence of significant differences between transgenic and non-transgenic cotton genotypes. Functional predictions of soil microbial communities indicated that microbial community function did not show significant differences between transgenic and non-transgenic cotton samples. These findings are essential for evaluating the environmental effects of transgenic crops and supporting the secure implementation of transgenic cotton.

## 1 Introduction

Cotton (*Gossypium hirsutum* L.) is an important fiber crop and oil source globally. The cotton yield accounts for about 30% of the world’s total yield every year in China (Sultana et al., 2023). More than 87% of the total Chinese cotton yield was produced in the Xinjiang-Uyghur Autonomous Region of China in 2020. Therefore, cotton is a very important crop in this region. The genetic engineering approach is widely used for crop improvement, especially for yield and resistance (James, 2000; Bacha and Iqbal, 2023). At present, more and more genetically modified (GM) crops are produced and planted commercially around the world, and their potential risks to the environment have attracted much attention (Nap et al., 2003; Angelo et al., 2022; Bacha and Iqbal, 2023).

Environmental safety assessments of GM crops are the main content of commercialization (Walker et al., 2003; Tran et al., 2018; Mandal et al., 2020; Angelo et al., 2022). Soil is an important place for material circulation and energy transformation in the whole ecosystem. Soil ecosystem stability directly affects crop growth and development, and ultimately affects the stability of the whole agricultural system. Many safety assessments have focused on the impact of GM crops on soil ecosystems (Shen et al., 2006; Shen et al., 2015). Transgenic crops may directly affect soil microbial and fungal communities, which in turn may lead to changes in agricultural soil ecosystems. Therefore, soil microbial communities are an important indicator of the safety of transgenic materials (Velmourougane and Sahu, 2013; Zhang et al., 2015a).

Plants affect soil mainly through “rhizo-deposition” and plant residues (Shahmoradi et al., 2019). Plants’ influence on the microbial population in the rhizosphere can be more than ten times greater than that in the bulk soil (Ali et al., 2013). This is because the root exudates and plant residues in the rhizosphere create a nutrient-rich and biologically active environment that promotes microbial diversity. Usually, the rich microbial diversity of the root is symbiotically associated with plants to promote better growth (Walker et al., 2003; Han et al., 2017). The soil microbial community of transgenic plants such as maize (*Zea mays*), soybean (*Glycine max*) (Sung et al., 2021),cotton (*Gossypium* sp.) (Shen et al., 2006), rice (*Oryza sativa* L.) (Wu et al., 2021) and tomato (*Solanum lycopersicum*) (Gao et al., 2015) were most studied. Most studies reported that transgenic crops had no or a minor effect on soil microorganisms and they had usually caused temporary changes in the root-rhizosphere microbiome, which disappear after a winter’s recovery. The influence of field management, soil properties, and season was greater than that of a transgenic event on soil microorganisms (Shen et al., 2006; Balachandar et al., 2008; Kapur et al., 2010; Velmourougane and Sahu, 2013). Only a few studies have shown that GM crops have significant effects on soil microorganisms (Nap et al., 2003; Griffiths et al., 2007). Studies have found that transgenic *Bt* cotton (overexpressing endotoxin from *Bacillus thuringiensis*) can be cultured with higher bacterial and fungal diversity (Donegan et al., 1995; Velmourougane and Sahu, 2013; Liang et al., 2018).

With the development of high-throughput sequencing approaches, metagenomics has been applied to study soil microorganisms. It breaks through the limitations of unculturable microorganisms (Brooks et al., 2015). High-throughput sequencing of fungal 18S rRNA and internal transcribed spacer (ITS) is most suitable because of high copy number of ribosomal DNA and because it is easy to be amplify and separate. Ribosomal DNA contains conserved regions in 18S, 5.8S, and 28S but has high variability in the ITS region, where the evolution rate is 10 times higher than in 18S rDNA. This makes ITS the preferred method for studying fungal community diversity (Marti et al., 2013). Meanwhile, whole genome sequencing and 16S rDNA sequencing are two main strategies to study the bacterial macrogenomics. 16S rRNA is located on the small ribosomal subunits of prokaryotic cells, including 10 conserved regions and 9 hypervariable regions, which is considered to be the most suitable index for bacterial phylogeny and taxonomic identification (Youssef et al., 2009; Fang et al., 2015; Shen et al., 2015).

In our previous studies, the moss aldehyde dehydrogenase ALDH21 gene from the desiccation-tolerant moss *Syntrichia caninervis* was cloned and transformed into the upland cotton variety Xinnongmian 1 to obtain transgenic cotton (Yang et al., 2016; Yang et al., 2019; Yang et al., 2023). Transgenic cotton is drought-tolerant, salt-resistant and disease-resistant to *Verticillium dahliae*, that can decrease cotton yield in Xinjiang’s arid land with limited water supply. However, environmental safety is an important index for transgenic crops, and the assessment of soil microorganisms is an important research direction for safety. In this research, we examined the chemical properties of the soil in cotton fields. We utilized high-throughput sequencing to evaluate the fungal and bacterial communities in soil after one growth period using ITS and 16S rRNA. Alpha and beta diversity were employed to describe the abundance and diversity of microbial communities in addition to exploring inter-sample correlations, species composition correlations with environmental factors, and microbial function predictions to assess the effects of transgenic material on soil microbial communities around the roots. Our objective was to evaluate the environmental safety of transgenic cotton and investigate potential changes in soil microbial populations due to transgenic cotton cultivation. Our findings confirmed that the cultivation of transgenic cotton did not cause significant ecological harm to these microbial communities.

## 2 Materials and methods

### 2.1 Experimental materials and design

Soil samples were collected from the artificial infection background of *ScALDH21* transgenic and non-transgenic cotton at the Manasi experiment station of the Xinjiang Academy of Agriculture Science. The Latin square design divided the cropping area into 9 plots where transgenic cotton lines 92, 96 (described as L1, L2, respectively) and non-transgenic cotton line 779 (described as NT) were planted, and each line had 9 repetitions. Since 2013, the same cotton lines have been continuously planted in the experimental plots used in this study. Bulk soil samples from the 5 centimeter periphery and 5-10 centimeter depth of cotton plants were collected after a growth season from the center of plots and stored at -80°C.

The soil chemical properties were detected in the Central Laboratory of Xinjiang Institute of Ecology and Geography, Chinese Academy of Sciences, based on the methods of forest soil nitrogen (LY/T 1228-2015), phosphorus in forest soil (LY/T1232-2015), potassium in forest soil (LY/T1234-2015), soil organic matter (NY/T1121.6-2006 Soil Testing, part 6), and soil pH (NY/T1121.6-2006 Soil testing, part 2).

### 2.2 Genomic DNA extraction and PCR amplification

Total soil genomic DNA was extracted with the FastDNA SPIN Kit for Soil (MP Biomedicals, Inc., CA). DNA concentration and purity level were monitored on a 1% agarose gel and a Nanodrop 2000 spectrophotometer, respectively. Based on the concentration, DNA was diluted to 1 ng µL^-1^ using sterile water. Specific primer pairs (515F: 5’-GTGCCAGCMGCCGCGGTAA-3’ and 806R: 5’-GGACTACHVGGGTWTCTAAT-3’) for the V4 region of 16S rRNA were used to identify bacterial diversity, and ITS (ITS5-1737F: 5’-GGAAGTAAAAGTCGTAACAAGG-3’and ITS2-2043R: 5’-GCTGCGTTCTTCATCGATGC-3’) was used to evaluate fungus diversity.

All PCR reactions were carried out in 30 µL reactions with 15 µL of Phusion® High-Fidelity PCR Master Mix (New England Biolabs). Thermal cycling consisted of initial denaturation at 98°C for 1 min, followed by 30 cycles of denaturation at 98°C for 10 s, annealing at 50°C for 30 s, and elongation at 72°C for 30 s. Finally, elongation was at 72°C for 7 min. The PCR products were purified with the GeneJETTM Gel Extraction Kit (Thermo Fisher Scientific). Sequencing libraries were generated using the Ion Plus Fragment Library Kit (Thermo Fisher Scientific) following the manufacturer’s instructions. The library’s quality was assessed on the Qubit@2.0 fluorometer (Thermo Fisher Scientific). Finally, the library was sequenced on an Ion S5TM XL platform, and 400/600 bp single-end reads were generated (Novogene company).

### 2.3 Sequencing, OTU cluster and species annotation

During analysis the barcodes and primers were removed from the raw sequencing data. After removing the barcode and primer sequences, the sample reads were merged using the FLASH software (V1.2.11, http://ccb.jhu.edu/software/FLASH/) (Magoc and Salzberg, 2011) to obtain Raw Tags. Then, quality control was performed on the Raw Tags using the fastp software to obtain high-quality Clean Tags. Finally, the Clean Tags with a database using the Usearch software to detect chimeric sequences and remove them (Haas et al., 2011), resulting in the final set of effective data known as Effective Tags.

The Effective Tags obtained above, was denoised with the DADA2 module using QIIME2 software (Li et al., 2020), and sequences with and abundance below 5 were filtered to obtain the final Amplicon Sequence Variants (ASVs) and feature table. Next, the classify-sklearn module in QIIME2 software was used align the obtained ASVs with a database in order to obtain species information for each ASV (Callahan et al., 2016). For the 16S regions, the Silva138.1 database (https://www.arb-silva.de/) was used and for the ITS region, used the UNITEv8.2 database (https://unite.ut.ee/).

Principal coordinate analysis (PCoA) and principal component analysis (PCA) analyses were performed using the vegan package in the R programming language (McMurdie and Holmes, 2013; Bro and Smilde, 2014). The vegan package provides specific functions, cmdscale and rda, that can be used to calculate PCoA and PCA. Non-metric multidimensional scaling (NMDS) analysis can be performed using the metaMDS function in the vegan package, which is capable of generating NMDS analysis based on the Bray-Curtis distance.

### 2.4 The analysis of microorganism diversity and complexity

Alpha diversity was assessed using five indices, including Chao1, Shannon, Simpson, ACE, and PD whole tree. QIIME2 was utilized for index calculation, while R software (version 2.15.3) was employed for dilution curve visualization and analysis of differences among groups based on the alpha diversity index. We utilized the Tukey HSD and Duncan methods of ANOVA to analyze the significance of differences between groups of the alpha diversity index.

Beta diversity was evaluated to determine differences in species complexity among samples. QIIME2 was employed to calculate the Unifrac distance and to construct a UPGMA sample clustering tree. Using R software (version 2.15.3). AMOVA, based on weighted Unifrac distance, was used to test for significant differences between the groups.

### 2.5 Correlation analysis of environmental factors

We conducted redundancy analysis to demonstrate the relationship between soil chemical properties and OTUs. The relative abundance data of fungal and bacterial communities at the OTU level were considered species data, while soil chemical data were viewed as soil environmental variables. The CANOCO5 software (Smilauer and Jan, 2014) was used to perform RDA analysis to evaluate the correlation between soil microbial communities and soil chemical factors. To assess differences in soil chemical data, ANOVA’s Duncan method was employed for analysis of variance and multiple comparisons. The CANOCO5 software was utilized to display the impact of environmental factors on microbial community structure. Given the nonlinear relationship between variables, we computed correlation coefficients between the two variables using the Spearman correlation analysis method, employing the psych package in the R programming language.

### 2.6 Function prediction

The FunGuild software was utilized to predict fungal functional profiles based on ITS sequencing data using the default database and settings. FAPROTAX (Louca et al., 2016) was used to predict bacterial functional profiles based on 16S rRNA sequencing data using the default database and settings.

## 3 Results

### 3.1 Soil chemical properties

**Figure 1:**
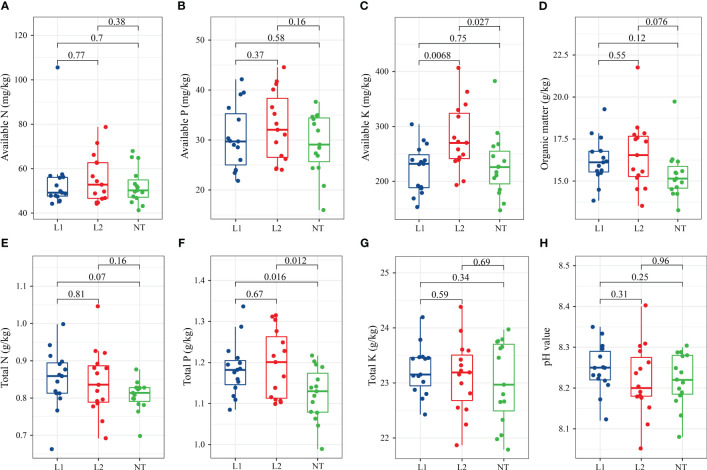
Soil chemical properties in cotton fields. **(A)** Available N (mg/kg) **(B)** Available P (mg/kg). **(C)** Available K (mg/kg). **(D)** Organic matter (g/kg). **(E)** Total N (g/kg). **(F)** Total P (g/kg). **(G)** Total K (g/kg). **(H)** pH value. The data were tested using the t-test, and a mean ± standard deviation was reported for each group with a sample size of n = 15 in each group. Statistical significance was expressed numerically, with a significance level of *P* < 0.05 indicating a significant difference between the two groups.

To determine chemical properties, soil samples were collected from 5 centimeters around the periphery and 5-10 centimeters deep. We measured eight soil chemical properties in plots planted with two transgenic lines (L1, L2) and non-transgenic cotton (NT). The analysis revealed that the levels of available nitrogen (N) (**Figure 1A**), available phosphorus (P) (**Figure 1B**), and available kalium (K) (**Figure 1C**) in the independent transformed line L2 were slightly higher compared to the transgenic line L1 and the non-transformed line NT (**Figures 1A–C**). The available N (**Figure 1A**) and available P (**Figure 1B**) content in each cotton line did not exhibit significant changes, while a significant difference was observed for available K (**Figure 1C**) between L2 and NT. The levels of soil organic matter in L1 (16.29 g/kg) and L2 (15.67 g/kg) were slightly higher than NT, although this was not significant (**Figure 1D**). Likewise, no significant difference was observed in total N and total K in the soil among the different cotton lines (**Figures 1E, G**). However, it should be noted that the total P content in L1 and L2 was significantly higher than that of NT (**Figure 1F**). The soil pH values of all cotton lines were found to be alkaline, exceeding 8, and there was no significant difference among the three groups (**Figure 1H**).

### 3.2 Sequencing results and quality control

**Figure 2:**
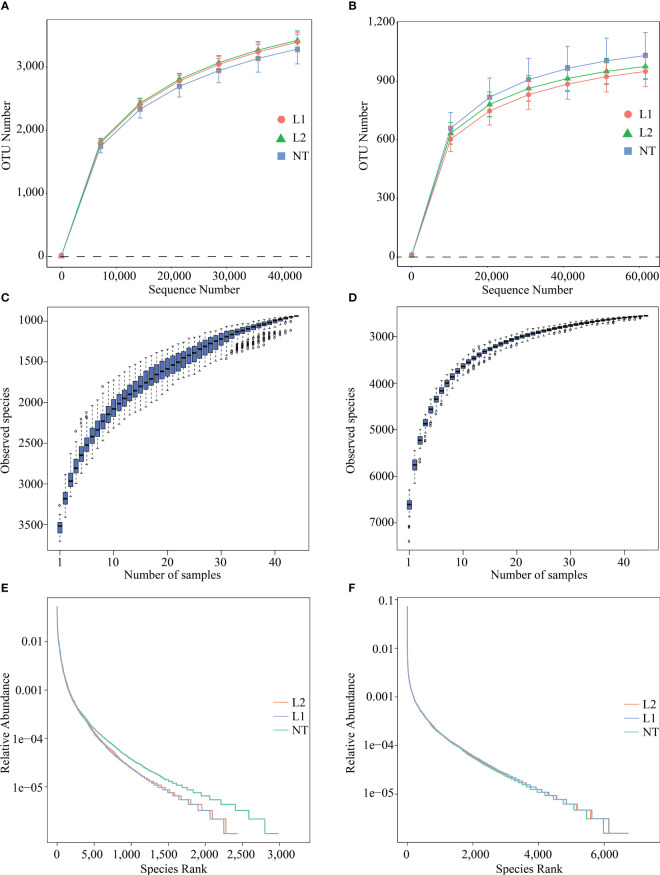
Species diversity analysis of sequenced samples. **(A)** Rarefaction Curve for fungal sequencing. **(B)** Rarefaction Curve for bacteria sequencing. **(C)** species accumulation boxplot of species sequenced by fungi. **(D)** species accumulation boxplot of species sequenced by bacteria. **(E)** Rank abundance curve for fungi sequencing. **(F)** Rank abundance curve for bacteria sequencing.

After filtering out low-quality and short sequence reads, a total of 81073.85 and 83378.48 raw reads were obtained from fungal and bacterial sequencing, respectively. Next, after the initial quality control process, on average 80165.93 and 78516.26 clean reads were obtained, respectively. The average length of fungal ITS was 227.3 bp and that of bacterial 16S rRNA was 417 bp. The sequencing quality was high, with a sequencing error rate less than 1% in clean reads. The operational transcriptional unit (OTU) clustering was performed with 97% consistency, and fungal sequencing yielded 3566 OTUs (**Table S1**), and bacterial sequencing yielded 7481 OTUs (**Table S2**). The rationality of total OTUs of each soil sample was detected by rarefaction curves. With the increase in sequencing depth, OTU numbers of fungi (**Figure 2A**) slowly increased, and the curve tended to be smooth. The rarefaction curves of L1, L2, and NT samples were consistent with this trend, and the sequencing depth of fungi was reasonable. The bacterial (**Figure 2B**) rarefaction curve was basically flat but still not saturated, and a small number of bacterial species may not be detected. The number of OTUs increases with the sample size, as depicted in the species accumulation boxplot (**Figures 2C, D**). The curve reaches stability as the number of OTUs saturates and fewer new OTUs are added to each sample, signifying the sequencing depth’s accuracy in representing fungal and bacterial composition. We used rank abundance curves to compare the differences in species composition between transgenic and non-transgenic cotton microbiomes. The results showed that, in different groups, the curve shapes of fungal communities were roughly similar but deviated at the same abundance threshold (**Figure 2E**), while the curve shapes of bacterial communities were also similar but did not deviate at the same abundance threshold (**Figure 2F**). In addition, the inflection points of the curves were analyzed, and they all appeared to have similar abundance values. The analysis of rank abundance curves indicated that the species composition and distribution patterns of root-associated microbiomes in transgenic and non-transgenic cotton were consistent.

### 3.3 Comparative analysis of multiple samples

**Figure 3:**
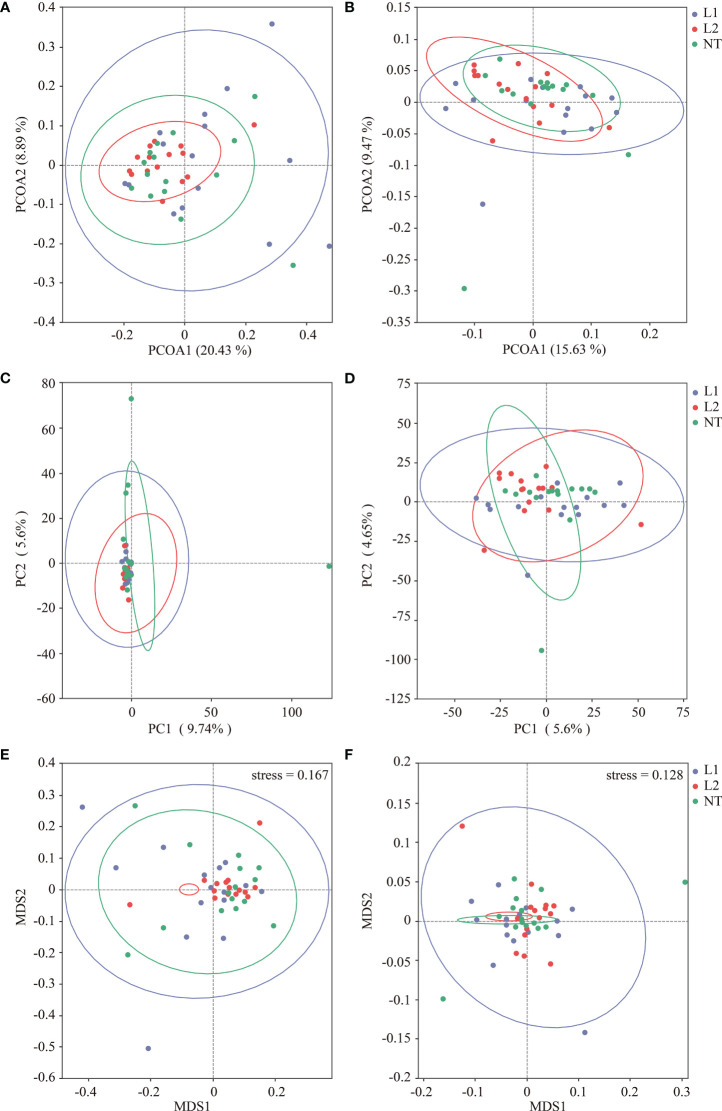
Multidimensional analysis of samples using PCOA, PCA, and NMDS. **(A)** fungi PCoA analysis. **(B)** Bacteria PCoA analysis. **(C)** fungi PCA analysis. **(D)** Bacteria PCA analysis. **(E)** fungi PCA analysis. **(F)** Bacteria PCA analysis.

The principal coordinate analysis (PCoA) was executed to explore the dissimilarity among microbial communities obtained from different soils. Variation in fungi was explained by 20.43% and 8.89% by PCoA1 and PCoA2, respectively (**Figure 3A**), while variation in bacteria was explained by 15.63% and 9.47% by PCoA1 and PCoA2, respectively (**Figure 3B**). PCoA analysis demonstrated the sample clustering of L1, L2, and NT, where soil samples from different groups clustered together, indicating a similarity in the microbial community structure between the different groups. We carried out principal component analysis (PCA) to evaluate the structural variances of fungal and bacterial communities in the different samples. PC1 and PC2 explained 9.74% and 5.6% of the total variation in fungi, respectively (**Figure 3C**), while PC1 and PC2 explained 5.6% and 4.65% of the total variation in bacteria, respectively (**Figure 3D**). PCA1 and PCA2 scatter plots showed no significant separation between the different groups, suggesting that no significant variation in fungal and bacterial community composition was observed between the samples. Non-metric multi-dimensional scaling (NMDS) was used to analyze the variability between fungal and bacterial communities at different ecological niches. MDS1 and MDS2 scatter plots indicated no significant variances in the fungal and bacterial communities of different groups and showed some similarity in their composition and relative abundance (**Figures 3E, F**).

### 3.4 Microbial diversity

**Figure 4:**
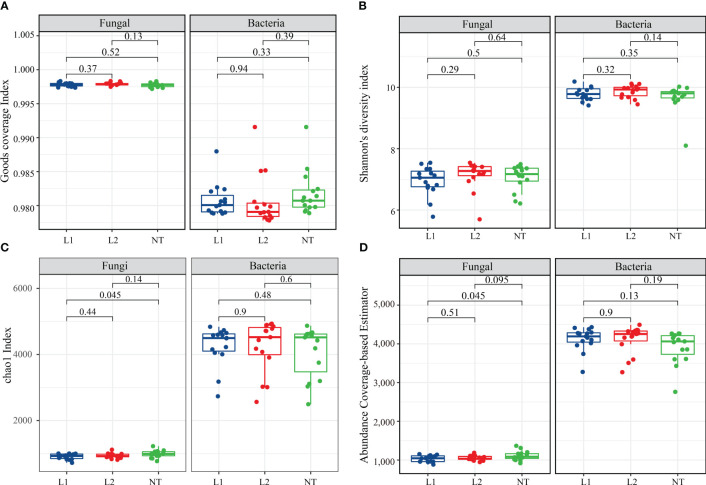
Alpha diversity index. **(A)** Goods coverage index for fungi and bacteria. **(B)** Shannon’s diversity index for fungi and bacteria. **(C)** chao1 Index for fungi and bacteria. **(D)** Abundance coverage-based estimator for fungi and bacteria. The data were tested using the t-test, and a mean ± standard deviation was reported for each group with a sample size of n = 15 in each group. Statistical significance was expressed numerically, with a significance level of *P* < 0.05 indicating a significant difference between the two groups.

In this study, we examined the alpha and beta diversity indices for fungi and bacteria in soil samples from transgenic (L1 and L2) and non-transgenic (NT) cotton. These indices reflect the abundance and diversity of fungi and bacteria in the soil. The coverage in all three samples exceeded 99.9%, indicating near-perfect coverage (**Figure 4A**). We used Shannon’s diversity index (Shannon), the Chao1 index (Chao 1), and the abundance Coverage-Based Estimator (ACE) indices, which are widely used in ecology to assess fungal and bacterial abundance. Our findings show that the Shannon and Chao 1 indices did not exhibit significant variation between the transgenic and non-transgenic cotton soils (**Figures 4B, C**). The ACE fungal level was slightly higher than L1 in NT but not different from L2 (**Figure 4D**). Beta diversity analysis revealed variability between L2 and NT and L1 in the fungal community, resulting in reduced diversity, while L2 exhibited reduced bacterial diversity, but overall, the magnitude of change was not significant (**Figure S1**). In conclusion, our findings suggest that there were no substantial changes in the diversity of soil microorganisms between transgenic and non-transgenic cotton samples.

### 3.5 Composition of soil microbial community

**Figure 5:**
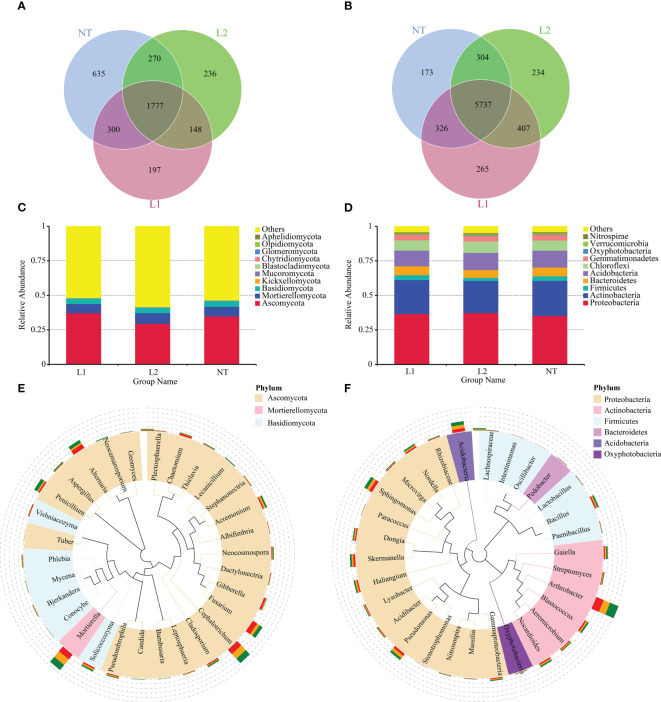
Species composition of the sample microbial community. **(A)** Venn diagram of samples based on fungal OTUs. **(B)** Venn diagram of samples based on bacteria OTUs. **(C)** Species composition at the fungal phylum level. **(D)** Species composition at the bacteria phylum level. **(E)** Species composition at the fungal genus level. **(F)** Species composition at the bacteria genus level.

The statistical analysis revealed that the L1, L2, and NT groups had 2422, 2431, and 2982 OTUs for fungal species, respectively, and 1777 OTUs were common to all groups (**Figure 5A**). In the case of bacterial OTUs, the statistical analysis revealed that L1, L2, and NT had 6726, 6682, and 6844 OTUs, respectively, and 5737 OTUs were common to all groups (**Figure 5B**). A statistical analysis was conducted at the phylum level for fungi and bacteria in the three groups. The results showed that the three groups had similar dominant species at phylum level in both fungi and bacteria. The fungal phyla *Ascomycota*, *Mortierellomycota*, *Basidiomycota*, *Kickxellomycota*, and *Mucoromycota* were widely distributed across all groups (**Figure 5C**). Similarly, *Proteobacteria*, *Actinobacteria*, *Acidobacteria*, *Chloroflexi*, and *Bacteroidetes* were relatively dominant among bacteria (**Figure 5D**). To conduct further investigation on the phylogenetic relationships and abundance at the genus level, representative sequences for the most abundant genera were aligned through multiple sequences, subsequently constructing a phylogenetic tree. The analysis of the bacterial and fungal genera in three groups, showed that the dominant genera were similar across all three groups, and all from the dominant phylum-level category. Specifically, *Mortierella*, *Cephalotrichum*, *Fusarium*, *Alternari*, and *Penicillium* were the main soil fungal genera (**Figure 5E**), while *Arthrobacter*, *Acidobacteria*, *Sphingomonas*, *Pseudomonas*, *Bacillus*, and *Skermanella* were the dominant soil bacterial genera (**Figure 5F**).

### 3.6 Screening of dominant species between different groups

**Figure 6:**
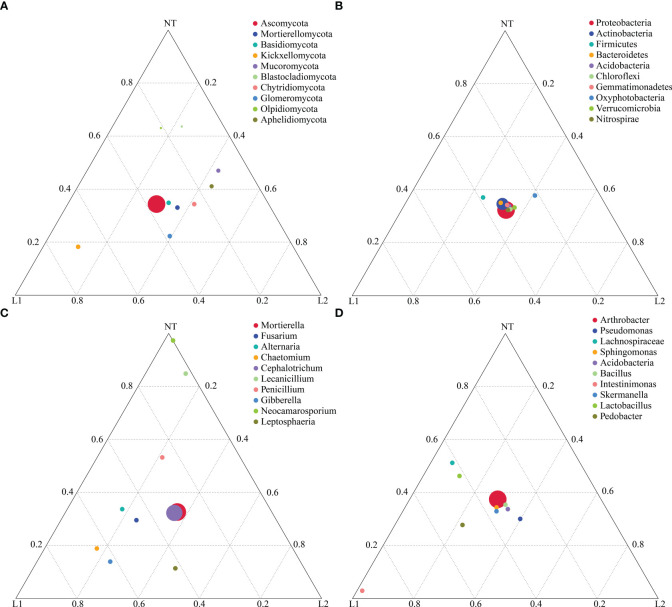
Highlighting dominant species on a ternary plot. **(A)** Fungal phylum level. **(B)** Bacteria phylum level. **(C)** Fungal genus level. **(D)** Bacteria genus level.

To uncover variations in dominant species among the three sample groups at different taxonomic levels (phylum, genus, and species), we identified the top 10 most abundant species for each level. These species were used to create a ternary plot, which allows for clear visualization of the differences in dominant species across the three sample groups at each taxonomic level. *Olpidiomycota* and *Blastocladiomycota* were found to be dominant in NT, whereas *Glomeromycota* and *Kickxellomycota* were more abundant in L1 and L2 (**Figure 6A**). In contrast, there were no significant differences in bacterial phyla abundance among the three groups (**Figure 6B**). At the fungal genus level, *Neocamarosporium* and *Lecanicillium* were found to be dominant in NT, while *Leptosphaeria*, *Gibberella*, and *Chaetomium* were found to be dominated in L1 and L2 (**Figure 6C**). The bacterial genera *Lachnospiraceae* and *Lactobacillus* showed dominance in relative abundance in NT, whereas *Intestinimonas* dominated in relative abundance in L1 and L2 (**Figure 6D**). Notably, there were no significant differences in *Bacillus* abundance among the three groups (**Figure 6D**). Fungal species including *Mortierella alpina*, *Fusarium delphinoides*, and *Cephalotrichum nanum* were identified as dominant among the three groups. Furthermore, *Neocamarosporium chichastianum* was found to have relative dominance in the group categorized as NT, while *Chaetomium longicolleum*, *Albifimbria verrucaria*, *Gibberella intricans*, and *Leptosphaeria sclerotioides* were found to have relative dominance L1 and L2 groups (**Figure S2A**). In addition, the bacterial communities were dominated by *Pseudomonas frederiksbergensis* shared among all three groups. However, *Lachnospiraceae bacterium* A4 showed dominant relative abundance in the NT group. Similarly, L1 and L2 groups, the *Lachnospiraceae bacterium* DW17, *Pedobacter duraquae*, and *Stenotrophomonas chelatiphaga* were also found to be dominant (**Figure S2B**).

### 3.7 Redundancy analysis of microbial and environmental factors

**Figure 7:**
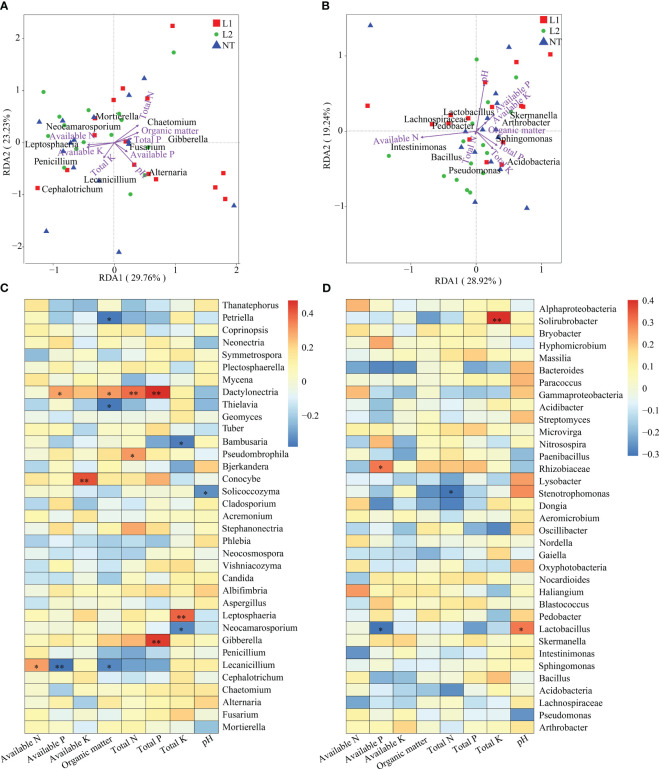
Environmental factor correlation analysis. **(A)** RDA of fungal genus-level diversity and environmental factors. **(B)** RDA of bacteria genus-level diversity and environmental factors. **(C)** Spearman analysis of fungal genus-level diversity and environmental factors. **(D)** Spearman analysis of bacteria genus-level diversity and environmental factors. In the heatmap, each cell’s color represents the correlation between two variables. * In the heatmap indicates a significance level of 0.05, and ** indicates a significance level of 0.01.

We investigated the correlation between microbial and environmental factors by conducting a redundancy analysis (RDA) of bacteria and fungi on a genus level. The RDA results indicated that 29.76% of the correlation was identified on axis 1 and 23.23% on axis 2 for the fungal genus level. The analysis indicates that available K had a prominent role in explaining axis 1, while pH had a crucial role in explaining axis 2. Among them, available K showed the highest impact on *Penicillium*, whereas pH had the most significant effect on *Alternaria* (**Figure 7A**). Our findings also revealed that the RDA analysis carried out at the bacterial genus level explained 28.92% on axis 1 and 19.24% on axis 2. The analysis identified that available P had the highest contribution in Axis 1, whereas total P had the greatest contribution to Axis 2. Available P had a more profound impact on *Acidobacteria*, whereas total P significantly impacted *Skermanella* (**Figure 7B**). Spearman correlation analysis and coefficients were employed in this study to determine the correlation between environmental factors and microbial species richness (alpha diversity). Spearman rank correlation was used to examine the inter-variation relationship between environmental factors and species. The purpose of this analysis was to obtain a correlation and determine significant differed values between the two variables. The abundance of fungal genera was significantly influenced by various environmental factors. Specifically, *Dactylonectria* was significantly affected by available P, organic matter, total N, and total P, whereas *Conocybe* abundance was significantly affected by available K. *Leptosphaeria* abundance was significantly influenced by total K, while *Gibberella* abundance was significantly affected by total P. In the case of *Lecanicillium*, available P had a significant impact on its abundance, while the abundance of *Solicoccozyma* was significantly influenced by pH (**Figure 7C**). At the bacterial genus level, total K had a significant impact on *Solirubrobacter* abundance, while *Rhizobiaceae* abundance was significantly affected by available P. Available P and pH had a significant impact on *Lactobacillus* abundance, whereas these environmental factors had no significant effect on *Bacillus* (**Figure 7D**).

### 3.8 Functional prediction of soil microbial community

**Figure 8:**
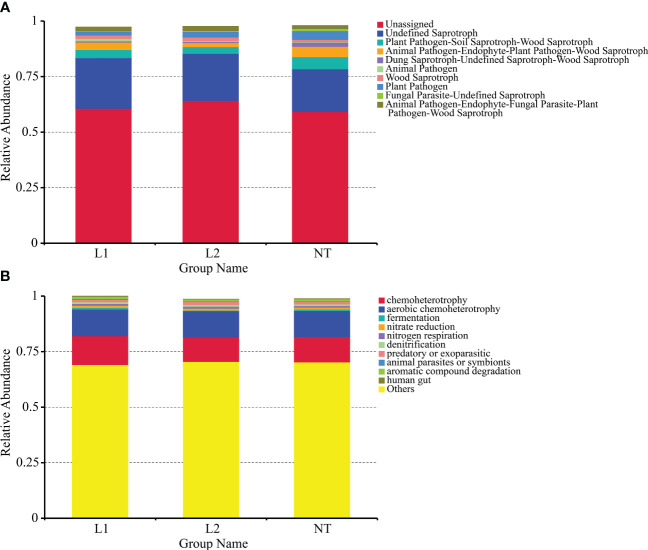
Functional prediction of soil microbial community based on metagenomic analysis. **(A)** Functional classification of fungi funguild in soils. **(B)** Functional classification of bacteria faprotax in soils.

We used the FUNGuild functional prediction software for ITS, and its prediction of guilds allowed us to study fungal functions from an additional ecological perspective. The results showed that the software could not predict the majority of fungal functions. The most abundant predicted functions were plant pathogen, soil saprotroph, and wood saprotroph, while animal pathogen was also prevalent (**Figure 8A**). We predicted the effects of bacterial biochemical cycling processes for different environmental samples using the FAPROTAX functional classification database based on species information. The results indicated that chemoheterotrophy had the highest abundance, and fermentation, nitrate reduction, nitrogen respiration, and denitrification also accounted for a significant proportion of them (**Figure 8B**). In conclusion, our functional predictions of soil microbial communities were quite similar between transgenic and non-transgenic cotton, with no significant differences in microbial community functions observed.

## 4 Discussion

The goal of this research was to evaluate the influence of *ScALDH21* transgenic cotton on soil microbial communities’ environments. Using high-throughput sequencing and data analysis, we investigated the impacts of transgenic and non-transgenic cotton on the soil microbes. Our analysis revealed no significant differences between the microbial composition of transgenic and non-transgenic cotton. These findings imply that there is no statistical distinction between microbial community compositions on soil of transgenic and non-transgenic cotton. The results provide insights that promote major environmental challenges associated with transgenic cotton and affirm the safety of *ScALDH21* transgenic cotton.

Different plant species tend to cultivate their own root microflora comprised of diverse microorganisms, which reflects the soil’s agricultural ecosystem. The soil surrounding the plant’s root system is usually divided into two parts: rhizosphere soil and bulk soil. The rhizosphere soil is a narrow area (~5 mm) that surrounds the plant’s root system, while the bulk soil extends beyond this distance. During the plant’s vegetative period, the soil microorganisms in this area are greatly affected by the “rhizosphere effect” compared with those in the bulk soil (Siciliano et al., 1998; Han et al., 2017; Shahmoradi et al., 2019; Vuong et al., 2020). Therefore, it is not suitable to evaluate the safety of transgenic plants within the rhizosphere soil. In this study, the environmental safety of transgenic crops was evaluated by selecting the bulk soil within 5 cm beyond the cotton root after the completion of the cotton-growing period.

The pH of the soil in all cotton fields exceeded eight, suggesting that the soil was alkaline and must have had a limiting effect on certain microbial populations. Previous studies have also shown that changes in bacterial communities are significantly correlated with pH (Li et al., 2018). Soil microorganisms are significantly affected by the effect of organic matter content in soil on saprophytic fungi (Setala and McLean, 2004). Soil chemical property analysis revealed that the soil in the cotton field exhibited alkaline properties. After one year, the content of organic matter in L1 and L2 was slightly higher compared to the non-transgenic cultivars. Significant differences were found only in the levels of available K and total P, both of which also showed significant increases. These findings suggest that the cultivation of all cotton cultivars did not have a significant adverse impact on the soil’s chemical properties. Furthermore, in comparison to other studies, these results are affected by species, plant breed, and geographic variation (Shen et al., 2006; Liang et al., 2018), which means that more research on these factors is needed.

Our sequencing results showed that species diversity of both fungi and bacteria was similar in both transgenic and non-transgenic cotton, which is consistent with previous studies indicating that transgenic *Bt* cotton has no significant effect on soil microbial communities (Shen et al., 2006; Hu et al., 2009; Kapur et al., 2010; Zhang et al., 2015b). We also performed a comparative analysis of multiple samples to compare the diversity and composition of soil microorganisms between transgenic and non-transgenic cotton samples. Our analysis of alpha and beta diversity indices revealed that there were no significant variations in the diversity of soil microorganisms between the two groups, supporting the notion that transgenic cotton expressing multi-resistant *ScALDH21* has negligible effects on soil microbial communities.

Previous studies have reported conflicting results on the impact of transgenic crops on soil microbial communities. Transgenic *Bt* rice lines produce a significant effect on the diversity and abundance of endophytic bacteria in leaves and roots, while only minor differences were found in the bacterial communities of the inter-root soils between *Bt* and non-*Bt* rice lines (Wu et al., 2021). Our results indicate that transgenic cotton expressing multi-resistant *ScALDH21* had negligible effects on the diversity and composition of soil microorganisms, which is consistent with previous results that transgenic crop cultivation had no effect on soil microorganisms (Liang et al., 2018; Guan et al., 2021; Chen et al., 2022; Xu et al., 2023). Interestingly, the transgenic ath-miR399d tomato line has an effect on microbial communities and diversity in soil over a short period of time (Gao et al., 2015), and this difference may be due to the different transgenic plants and microbial communities investigated in these studies. We also used ternary plots to visualize the differences in the abundance of dominant genera between the groups. We found that the difference in abundance of dominant genera between transgenic and non-transgenic cotton was small but not negligible. There were some genera in relative abundance in both transgenic and non-transgenic cotton samples that were different, such as *Leptosphaeria*, *Intestinimonas*, and *Pedobacter*, suggesting that these genera may be more sensitive to the transgenic cotton. Overall, our findings indicate that transgenic cotton expressing multi-resistant *ScALDH21* has minimal impact on the diversity and composition of soil microorganisms, which is consistent with previous studies on *Bt* cotton. However, our study also provides further insights into the abundance of dominant genera in transgenic and non-transgenic cotton, which may have important implications for monitoring potential ecological risks associated with the widespread use of transgenic crops.

To assess the environmental safety of transgenic cotton expressing *ScALDH21* gene, we analyzed the diversity and composition of soil microorganisms and their relationship with environmental factors. Our RDA analysis showed a significant association between soil chemical properties and soil microbial community composition, which is consistent with previous studies (Xu et al., 2023). However, we found that no significant differences were seen in different cotton lines. Although numerous studies have investigated the effects of environmental factors on soil microbial communities, few have explored the effects of transgenic cotton on these communities. Our results suggest that transgenic cotton expressing multi-resistant *ScALDH21* did not affect the correlation between microbial and environmental factors compared to non-transgenic cotton. Similarly, previous studies found no significant difference in the relationship between microbial composition and environmental factors in soils planted with transgenic *Bt* cotton and non-transgenic cotton (Hu et al., 2009). This finding suggests that the nutrient availability in soil has a significant impact on the fungal community. In conclusion, our RDA analysis demonstrated that there was no significant difference in the correlation between microbial composition and environmental factors between transgenic and non-transgenic cotton. These findings provide essential insights into the environmental safety of transgenic cotton expressing multi-resistant *ScALDH21* and support the safe implementation of transgenic cotton. Nonetheless, more studies are required to determine the long-term effects of transgenic crops on soil microbial communities and their relationships with environmental factors.

The functional prediction results demonstrated that there were no notable differences in microbial community function between the three groups. Our study provides further evidence that the use of transgenic crops expressing multi-resistant *ScALDH21* has minimal effects on soil microbial communities, similar to *Bt* cotton (Shen et al., 2006). Fungi and bacteria play critical roles in soil nutrient cycling and plant growth. As such, we used functional prediction analysis to investigate the functional diversity of the soil microbial community based on the abundance of genus-level OTUs. Our results revealed no significant differences in microbial community function between transgenic and non-transgenic cotton samples. The consistency between the microbial functional profiles suggests that transgenic cotton expressing multi-resistant *ScALDH21* does not adversely affect the activities of soil microorganisms.

Our study results provide insights into the environmental safety of transgenic cotton expressing multi-resistant *ScALDH21* and support the safe implementation of these crops. However, the long-term effects of transgenic crops on soil microbial communities remain unclear. Therefore, long-term studies are required to ensure the environmental sustainability of transgenic crops.

## 5 Conclusion

In this study, the impact of ScALDH21 transgenic cotton on soil microbial communities was assessed using high-throughput sequencing technology. The results showed that there were no significant differences in the diversity and composition of soil microorganisms between transgenic and non-transgenic cotton. Environmental factors such as soil pH, available K, available P and total P had a significant impact on microbial community composition and species richness. Analysis also revealed that the dominant phylum, genus, and species were similar across all three groups. Overall, these findings demonstrate the feasibility of *ScALDH21* transgenic cotton from an environmental safety perspective and provide important insights for the safety evaluation of transgenic crops.

## Data availability statement

The original contributions presented in the study are included in the article/**Supplementary Material**. Further inquiries can be directed to the corresponding authors.

## Author contributions

All authors contributed to the study’s conception and design. DYZ and HY conceived of and designed the experiments. QY, JW, and HF performed the experiments and wrote the articles. DWZ, HF and HY helped to perform the experiments and collected the data. QY and JW participated in the statistical analysis. TB helped with the chart processing. QY, HY, TB and DYZ contributed to manuscript discussion and revision. All authors contributed to the article and approved the submitted version.
