# Supplementary figures and images for: Effects of multi-resistant ScALDH21 transgenic cotton on soil microbial communities
Source: Front Microbiomes. 2023 Oct 3;2:1248384. doi: 10.3389/frmbi.2023.1248384 (PMC12993657; doi:10.3389/frmbi.2023.1248384)

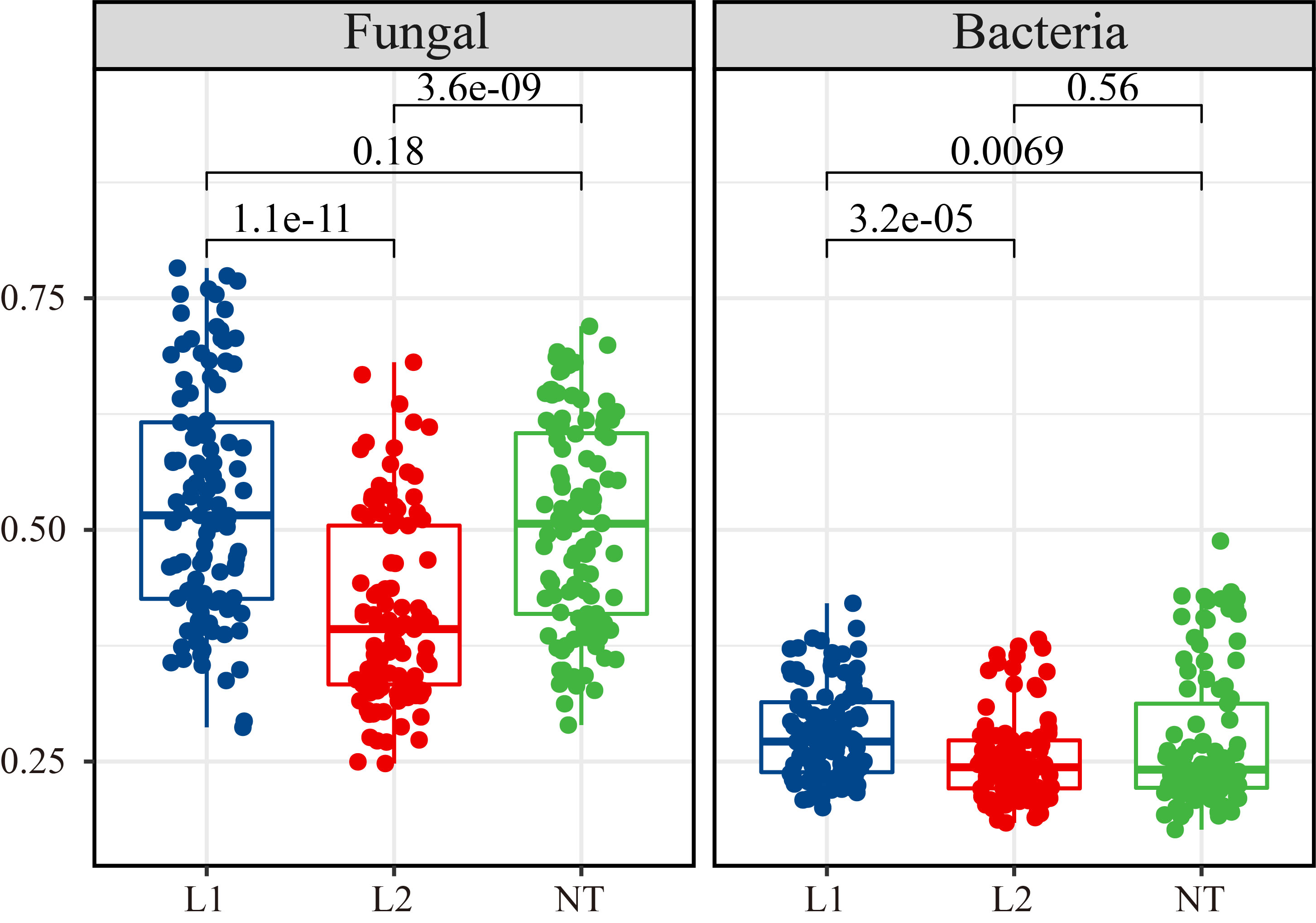

Supplement: Supplementary file 1 [file Image_1.jpeg]

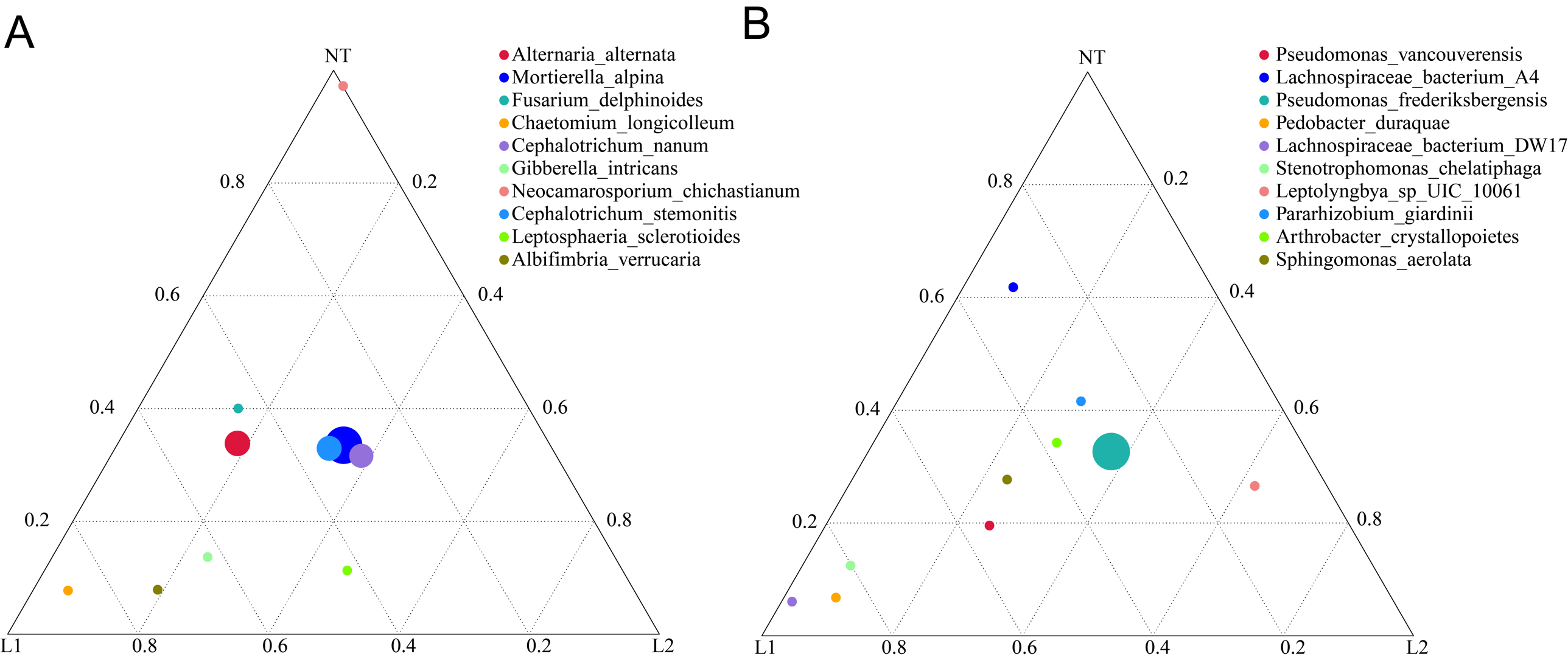

Supplement: Supplementary file 2 [file Image_2.jpeg]
