# Supplementary material for: Effects of multi-resistant ScALDH21 transgenic cotton on soil microbial communities
Source: Front Microbiomes. 2023 Oct 3;2:1248384. doi: 10.3389/frmbi.2023.1248384 (PMC12993657; doi:10.3389/frmbi.2023.1248384)
Supplement: Supplementary file 3 [file Table_1.docx]

**SUPPLEMENTARY DATA**

**Table S1.** Fungal OTUs summary table.

**Table S2.** Bacteria OTUs summary table.

**Figure S1.** Beta diversity index.

**Figure S2.** Highlighting dominant species on a ternary plot. (A) Fungal species level. (B) Bacteria species level.
